# MicroRNAs expression profile in solid and unicystic ameloblastomas

**DOI:** 10.1371/journal.pone.0186841

**Published:** 2017-10-20

**Authors:** A. Setién-Olarra, X. Marichalar-Mendia, N. G. Bediaga, P. Aguirre-Echebarria, J. M. Aguirre-Urizar, A. Mosqueda-Taylor

**Affiliations:** 1 Oral Medicine and Pathology, Department of Stomatology II, University of the Basque Country (UPV/EHU), Leioa, Spain; 2 BIOMICs Research Group, Lascaray Research Center, University of the Basque Country (UPV/EHU), Miguel de Unamuno, Vitoria-Gazteiz, Spain; 3 Head and Neck Section. Service of Pathology. University Hospital of Donostia. University of the Basque Country/EHU. San Sebastian; 4 Health Care Department, Universidad Autónoma Metropolitana Xochimilco, Ciudad de México, México; University of South Alabama Mitchell Cancer Institute, UNITED STATES

## Abstract

**Objectives:**

Odontogenic tumors (OT) represent a specific pathological category that includes some lesions with unpredictable biological behavior. Although most of these lesions are benign, some, such as the ameloblastoma, exhibit local aggressiveness and high recurrence rates. The most common types of ameloblastoma are the solid/multicystic (SA) and the unicystic ameloblastoma (UA); the latter considered a much less aggressive entity as compared to the SA. The microRNA system regulates the expression of many human genes while its deregulation has been associated with neoplastic development. The aim of the current study was to determine the expression profiles of microRNAs present in the two most common types of ameloblastomas.

**Material & methods:**

MicroRNA expression profiles were assessed using TaqMan® Low Density Arrays (TLDAs) in 24 samples (8 SA, 8 UA and 8 control samples). The findings were validated using quantitative RTqPCR in an independent cohort of 19 SA, 8 UA and 19 dentigerous cysts as controls.

**Results:**

We identified 40 microRNAs differentially regulated in ameloblastomas, which are related to neoplastic development and differentiation, and with the osteogenic process. Further validation of the top ranked microRNAs revealed significant differences in the expression of 6 of them in relation to UA, 7 in relation to SA and 1 (miR-489) that was related to both types.

**Conclusion:**

We identified a new microRNA signature for the ameloblastoma and for its main types, which may be useful to better understand the etiopathogenesis of this neoplasm. In addition, we identified a microRNA (miR-489) that is suggestive of differentiating among solid from unicystic ameloblastoma.

## Introduction

Odontogenic tumors (OT) represent a specific pathological category that is particularly interesting due to its unique and complex etiopathogenesis that causes lesions with variable, sometimes unpredictable biological behavior. Although most OT are benign, some may exhibit local aggressiveness and high recurrence rates [1,2]. One of such tumors is the ameloblastoma, a neoplasm composed by proliferating odontogenic epithelium that resembles the enamel organ, which may produce diverse clinical and histomorphological variants [2]. The most common type of amelobastomas is the solid/multicystic (SA), but there is also an enterely cystic variant, the unicystic ameloblastoma (UA), which is considered a much less aggressive entity as compared to SA [1,2] and should therefore be identified and treated in a less aggressive way.

To date, most molecular studies on ameloblastomas have focused largely on the search for markers [3,4], as well as on the presence of genetic alterations [5–7], and, to a lesser extent, on the epigenetic alterations present in these neoplasms [8,9] that helps to ensure diagnosis and better define its prognosis. Recently, it has been shown that a hyperactive RAS–RAF–MAPK pathway is closely associated with the pathogenesis of the ameloblastoma, either through EGFR-mediated signalling or through frequent activating mutations in the BRAF gene [10].

MicroRNAs are small non-coding RNA molecules (21 to 25 base pairs in length) that regulate post-transcriptional gene expression. The microRNAs bind partially to the 3' region of the messenger RNA (mRNA) causing transcriptional repression or direct degradation of the mRNA [11]. MicroRNAs are known to regulate some basic cellular biological processes, such as growth, differentiation and cell death [12]; therefore, they may play an important role in neoplastic development, acting as both oncogenes and suppressor genes [13].

Different studies in oral cancer [14,15] report differences between tumoral and normal tissues when analyzing the expression profiles of microRNAs. In addition, changes in the expression profiles of microRNAs during malignant transformation of oral precancerous lesions have also been described [16,17]. However, studies that analyze the role of alterations of microRNAs in the development and progression of ameloblastomas are scarce [18].

The purposes of this study were to determine the expression profile of microRNAs present in the two main types of ameloblastoma, the solid and the unicystic types, and to validate the results in a set of independent samples.

## Material and methods

We studied oral samples of formalin-fixed and paraffin-embedded biopsies (FFPE) of 70 patients diagnosed with SA (27 cases), UA (16 cases), and 27 cases of dentigerous cyst (DC). These samples were obtained from the Oral Pathology Laboratory of the Metropolitan Autonomous University (Mexico) and the Oral and Maxillofacial Pathology Laboratory of the University of the Basque Country (EHU). In the screening phase of the SA group, a total of 8 patients (mean age, 30 years; SD, 12.2; female to male ratio, 1: 1) were included, whereas in the validation phase 19 patients (mean age, 31.9 years; SD, 15.2; female to male ratio, 0.9: 1) were included. Eight patients with UA were included in the screening phase (mean age, 18.9 years, SD, 8.7, female-to-male ratio, 1: 1), and the other eight cases were studied in the validation phase (mean age, 19.9 years; SD, 6.7, female-to-male ratio, 1: 1). Finally, 27 patients diagnosed with DC were included as a control group. In the screening phase, there were 8 cases (mean age, 36 years; SD, 17.9; female-to-male ratio, 1: 1), while 19 were included in the validation phase (mean age, 46.7 years; SD, 14.5; female -to-male ratio, 0.46: 1). The dentigerous cyst was chosen as a control because it is a non-neoplastic odontogenic cystic lesion that presents an epithelial component derived from the reduced epithelium of the enamel organ which is included in the differential diagnosis of UA both clinically and at a microscopic level in spite of its different biological behavior [19].

Table 1 shows the salient demographic characteristics of the three groups of lesions. In all cases, the most representative areas of each lesion were chosen for study.

**Table 1:** Demographic characteristics of the patients included in this study.

		Screening (n = 24)			Validation (n = 46)		
		Control (n = 8)	UA (n = 8)	SA (n = 8)	Control (n = 19)	UA (n = 8)	SA (n = 19)
Gender n (%)	Male	4 (50)	4 (50)	4 (50)	13 (68.4)	4 (50)	10 (52.6)
	Female	4 (50)	4 (50)	4 (50)	6 (31.6)	4 (50)	9 (47.4)
Age(years) Mean (SD)		36 (17.9)	18.9 (8.7)	30 (12.2)	46.7 (14.5)	19.9 (6.7)	31.9 (15.2)

This study was carried out following the principles of the Declaration of Helsinki on Ethical Principles for Medical Research Involving Human Subjects and was approved by the Ethics Committee for Research of the University of the Basque Country / EHU (CEIAB / 204/2015). The study followed a similar methodology to that described by Setién-Olarra et al. [17].

### RNA isolation

The total RNA, in which the microRNA was included, was extracted from sections of the paraffin blocks, which were previously dewaxed with xylol using the miRNeasy FFPE kit (Qiagen, UK). The purity and concentration of the RNA were determined by the OD260 / 280/230 readings using a NanoDrop ND-1000 spectrophotometer (Thermo Fisher Scientific, Eugene, OR, USA). The integrity of the RNA was determined by fluorometric quantification using the Qubit 3 Fluorometer (Life Technologies, Foster City, CA, USA).

### MicroRNA profiling

Expression profile analysis of microRNAs was performed in 24 samples (8 AS samples, 8 AU samples, and 8 control samples) using TaqMan® Low-Density Arrays (TLDAs) (Applied Biosystems, USA). In summary, the total RNA (150 ng) was converted to specific cDNA derived from mature microRNAs using Megaplex ™ Primer Pools A and B (Applied Biosystems), followed by a pre-amplification step using Megaplex Pre-amp Primers Pools A and B. Finally, a total of 9 μl of the preamplifier product was loaded onto TaqMan® Array Human MicroRNA Cards. A 7900 real-time RT-PCR System (Applied Biosystems) was used to run the assay.

### MicroRNA quantitative RT-PCR

Subsequently, we validated the microRNAs identified in an independent set of 91 oral samples (FFPE) (27 SA, 16 UA and 27 DC) by RT-qPCR using TaqMan® microRNA assays (Applied Biosystems).

Each microRNA was analyzed in triplicate and furthermore non-template RT controls were carried out for each experiment. The Ct values of the target microRNAs were normalized with the three normalizing microRNAs (miR-222, miR-17 and miR-106a), and the expression levels of each microRNA [relative amount (RQ)] were calculated using the method Comparison of Ct: Ct_SAMPLE_-Ct_MEANNORMALIZERS_.

### Data analysis

The data were analyzed using the software packages Sequence Detection System (SDS) and Expression Suit (Life Technologies) as previously described by Bediaga et al. [20]. The Ct values were determined using an automatic baseline and a threshold of 0.1. Valid data were imported into the HTqPCR package [21] for quality assessment, normalization, and statistical significance tests of Ct values between different groups. The data were normalized using the geometric mean of the entire expression of the set of microRNAs.

The microRNAs that showed a normalized performance similar to the expression value of the geometric mean were proposed as normalizer candidates for the subsequent validation phase, as previously described by Marabita et al. [22]. The miR-222, miR-17, and miR-106a microRNAs proved to be the best set of normalizers, confirming their suitability as endogenous normalizers with the algorithm software [23]. The difference in expression of the microRNAs found between the different groups was further evaluated by empirical Bayes moderated t-tests. All p-values obtained were adjusted by the Benjamini-Hochberg procedure. We selected the microRNAs that showed a two-fold difference in the Fold Change value [Fold Change (FC)> 2] and an adjusted p-value <0.05 between SA vs normal and UA vs normal “S1 Table”. For the identification of the different subgroups defined by expression profiles of microRNAs, unsupervised cluster analyses were performed using the Euclidean distance between the Ct values. On the other hand, the paired comparisons of the quantitative data obtained in the RT-qPCR experiments were performed using the Mann-Whitney test. All tests were two-tailed, and p-values <0.05 were considered statistically significant. Statistical analyzes carried out in the validation phase were performed using SPSS v18.

## Results

### Identification of aberrant expression profiles of microRNAs in SA and UA

**Fig 1:**
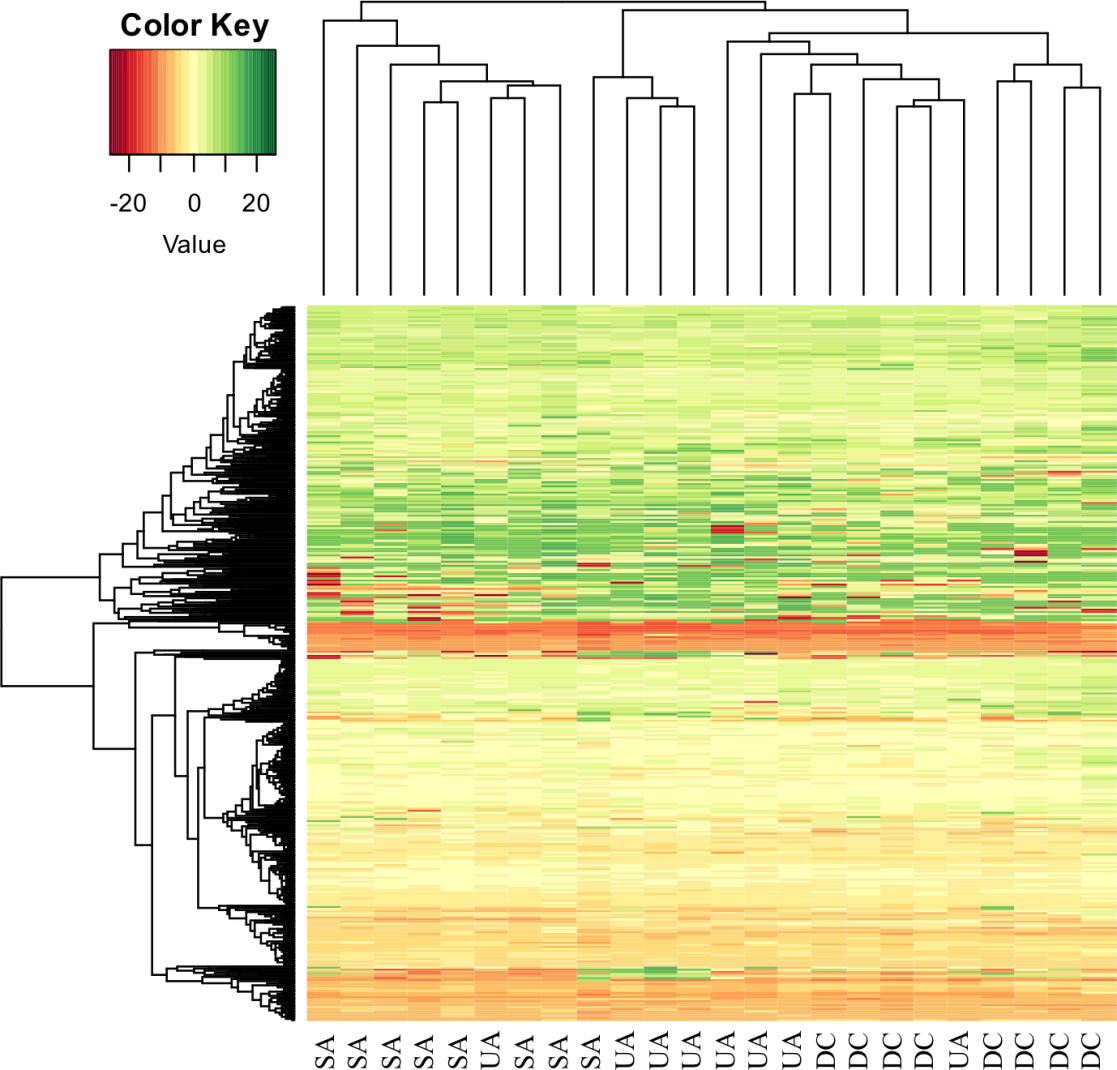
Unsupervised hierarchical clustering analysis using the differentially expressed microRNAs separates SA, UA, and control tissues. The heatmap (Euclidean distance) represents the delta-Ct values. Color heatmap correspond to microRNA expression as indicated in the color key: red (underexpressed) and green (overregulated).

The expression profile of 768 mature miRNAs was analyzed in a total of 24 samples. Of these, 56 were present in SA and 20 in UA. Visual approximation analyses based on unsupervised clusters of these microRNAs resulted in a differentiated expression profile among the 3 groups of lesions (Fig 1).

**Fig 2:**
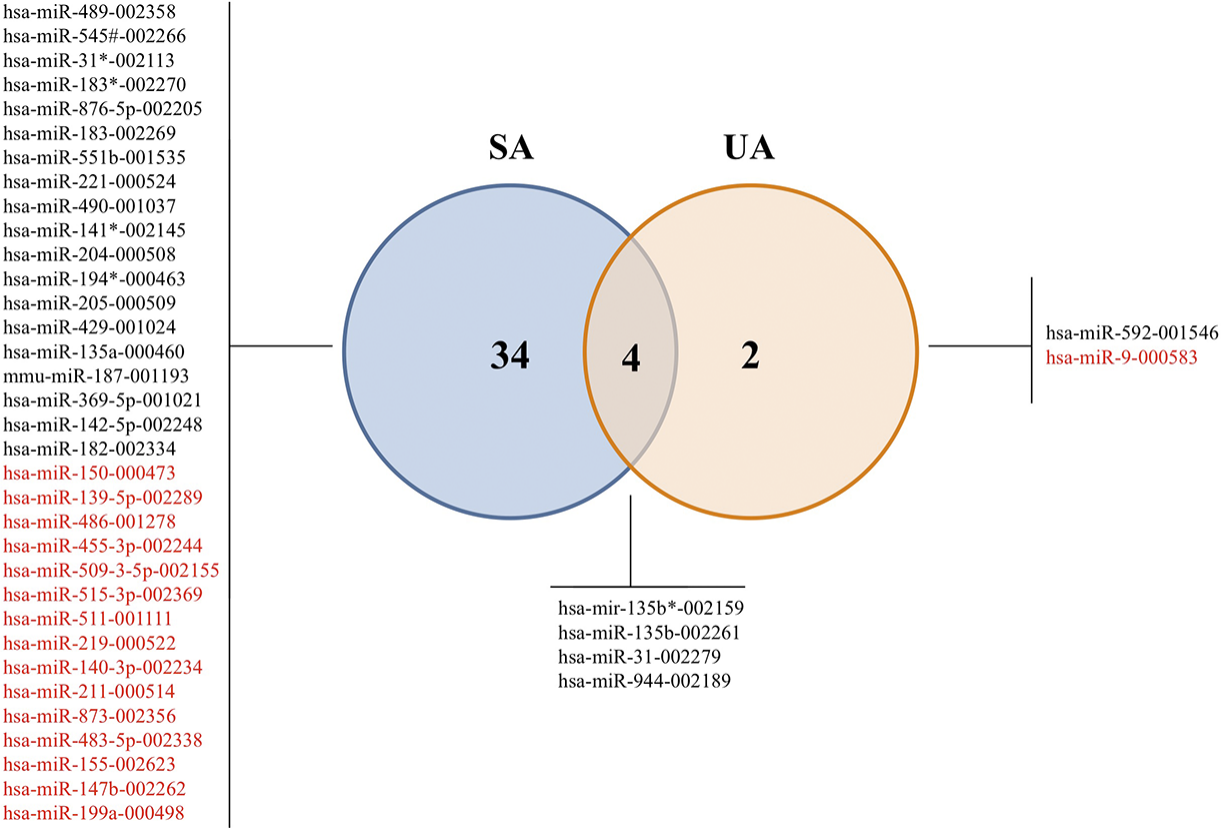
A Venn diagram showing microRNA signatures in ameloblastoma types. Significantly deregulated microRNAs (adjusted p-value <0.05 and FC ≤0.5 or ≥2) within the two groups compared with control group. Upregulated in black and downregulated in red color.

Next, we proceeded to analyze which miRNAs were differentially expressed in each group (SA and UA) compared to the control. Using a significance level of p <0.05 (adjusted for multiple tests) and a FC ≤ 0.5 or ≥ 2 cutoff, 40 microRNAs were classified as dysregulated in the ameloblastoma samples. Specifically, 38 microRNAs were classified as dysregulated in the SA group (23 overexpressed and 15 underexpressed) and 6 in the UA group (5 overexpressed and 1 underexpressed). In turn, both groups shared 4 unregulated miRNAs versus the control group (Fig 2).

The 13 microRNAs mostly deregulated among the SA and the control group and among the UA and the control group were selected for a posterior validation in an independent set of samples.

### Validation of microRNAs expression profiles between UA and SA groups

After applying the inclusion criteria (|FC| <0.2 or> 5 and p adjusted <0.05), as previously mentioned, biological validation was performed by RT-qPCR of the 13 differently regulated miRNAs (hsa-miR-9, hsa-miR-135b*, hsa-miR-194*, hsa-miR-489, hsa-miR-592, hsa-miR-369-5p, hsa-miR-876-5p, hsa-miR-31, hsa-miR-135b, hsa-miR-211, hsa-miR-944, hsa-miR-142-5p, hsa-miR-455-3p), in an independent set of 46 samples corresponding to 19 SA, 8 UA and 19 controls.

When analyzing the expression of each miRNA, we observed statistically significant differences in 6 microRNAs among the UAs and the control group, (p <0.05) (hsa-miR-135b *, hsa-miR-592, hsa-miR-31, hsa-mir-135b, hsa-miR-944, hsa-miR-142-5p)). On the SA group, we observed differences in the expression between SAs and the control group in seven out of the 13 microRNAs selected for biological validation (p <0.05), (hsa-miR-135b *, hsa-miR- 489, hsa-miR-592, hsa-miR-369-5p, hsa-miR-31, hsa-mir-135b, hsa-miR-944). When comparing the SA group with the UA group, we only found statistically significant differences for microRNA-489 (p = 0.016) (Table 2).

**Table 2:** Biological validation of the 13 microRNAs selected.

microRNA	Chromosome		Unicystic Ameloblastoma vs Control		Solid Ameloblastoma vs Control		Solid Ameloblastoma vs Unicystic	
			FC	p value	FC	p value	FC	p value
hsa-miR-135b*	1		**8.52**	**<0.001**	**15.87**	**<0.001**	1.86	0.283
hsa-miR-944	3		**5.72**	**<0.001**	**8.91**	**<0.001**	1.56	0.333
hsa-mir-135b	1		**4.58**	**<0.001**	**5.67**	**<0.001**	1.24	0.585
hsa-miR-31	9		**3.59**	**<0.001**	**6.65**	**<0.001**	1.85	0.051
hsa-miR-592	7		**2.99**	**0.001**	**4.11**	**<0.001**	1.20	0.515
hsa-miR-142-5p	17		**1.55**	**0.006**	1.11	0.133	0.72	0.26
hsa-miR-369-5p	14		0.79	0.333	**0.66**	**0.015**	0.84	0.856
hsa-miR-489	7		0.85	0.449	**0.43**	**<0.001**	**0.50**	**0.016**
hsa-miR-876-5p	9		24.31	0.055	1.53	0.163	0.55	0.836
hsa-miR-194*	1		1.58	0.669	1.30	0.503	0.83	0.644
hsa-miR-211	15		1.27	> 0.05	1.01	0.825	0.79	0.881
hsa-miR-9	1		0.99	0.929	0.73	0.307	0.74	0.407
hsa-miR-455-3p	9		0.98	0.735	0.83	0.402	0.85	0.821
			

FC: Fold Change; Statistically significant values are represented in bold.

When analyzing the expression levels of the microRNAs selected for validation (Table 2), we observed that 5 of them (hsa-miR-135b *, hsa-miR-592, hsa-miR-31, hsa-mir-135b, hsa-miR-944) have similar patterns of expression, i.e., there are statistically significant differences between the UAs and the control group. There are also statistically significant differences between the SAs and control group; however, when comparing the levels of expression of these microRNAs between the UA and the SA groups, we did not find statistically significant differences.

## Discussion

Ameloblastoma is a benign odontogenic tumor that can exhibit a variably aggressive biological behavior as evidenced by its infiltrative growth and marked tendency for recurrence [1,2]. Interestingly, the main variants of this neoplasm (SA and UA) show clear differences on its prognosis, urging for a different surgical approach for each type, with less invasive procedures being adequate to control most UA. However, sometimes it can be difficult to make a precise pre-therapeutic diagnosis, so it may be important to elucidate if there are differences in the molecular pathways that are involved in their development that could be of assistance in differentiating among them [3]. Therefore, the search for new diagnostic and prognostic tools that help improve the characterization of ameloblastomas and provide us with new therapeutic strategies is a very important issue.

This study analyzes the expression profiles of micro-RNAs in ameloblastoma, and shows that this neoplasm has aberrant expression profiles of microRNAs that may pose diagnostic value.

In the screening phase we identified 40 microRNAs differently expressed in ameloblastoma. In addition, we have observed a clear progression of alterations in the expression of microRNAs from UA (6 altered microRNAs) to SA (38 altered microRNAs), 4 of which are shared by both types of ameloblastoma. After validation, we recognized significant differences in 6 microRNAs in relation to UA, 7 to SA and in 1 (miR-489) in relation to both tumor types. We consider that these results demonstrate a specific profile of altered microRNAs for these neoplasms, and the existence of a microRNA (miR-489) that is suggestive of differentiating between the two major types of ameloblastoma.

MicroRNAs with a differential expression in both SA and UA when compared to the control group are the microRNA miR-135b, miR-135b *, miR-31, miR-592 and miR-944.

It has been previously described [24] that miR-135b is a regulator of mineralization in the process of osteoblastic stem cell differentiation. In addition, a serum increase of miR-135b was recognized in patients with multiple myeloma who present bone lesions, a finding that could help to identify these patients [25]. There are also several studies [26,27] that linked deregulation of miR-135b with the progression of some malignant neoplasms, such as squamous cell carcinoma of the head and neck. Its overexpression would lead to an increase in cell proliferation, migration and the formation of cell colonies [26]. Recently, Nezu et al. [27] identified miR-135b as a key regulator in myxoid liposarcoma, the overexpression of which would favor neoplastic invasion and metastasis through the direct suppression of thrombospondin 2 (THBS2), a protein that mediates cell-extracellular matrix interactions. Furthermore, Jin et al. [28] linked miR-135b with the stimulation of osteosarcoma recurrence and lung metastasis via Notch and Wnt/β-Catenin signaling. Based on this information, we consider that overexpression of miR-135b observed in ameloblastoma could be related to tumor growth and regulation of osteogenesis.

In relation to miR-31, studies indicate that it would play an important role in the regulation of osteogenesis [29,30]. Recently, Weilner et al. [29] identified it as a crucial component during the inhibition of the osteogenic process, recognizing elevated plasma levels of this microRNA in patients with osteoporosis [29]. Furthermore, this microRNA could regulate osteogenic differentiation by its direct binding to the STAB2 gene, which codes for a transmembrane receptor that, in turn, regulates important processes such as angiogenesis and cell adhesion [30]. In addition, miR-31 would also participate in different neoplastic processes, including oral squamous cell carcinoma [31,32]. Siow et al. [31] indicated that its overexpression would be related to the tumor stage, with a special relevance in the early stages, while others described [32] that the activation of the EGFR-AKT-CEBPb pathway would promote overexpression of miR-31 in oral cancer. Finally, it is important to mention that different authors [33–35] described a direct association between the altered expression of miR-31 and the RAS-RAF-MAPK pathway, resulting in cell growth and cell survival, and which is closely associated with ameloblastoma pathogenesis, lung cancer [34] and colorectal cancer [33,35]. Edmonds et al. (2016) determined that miR-31 was not only highly overexpressed in lung adenocarcinoma but also significantly correlated with patient survival. This microRNA is capable of promoting lung cancer by subexpressing several regulators (SPRED1, SPRED2, SPRY1, RASA1, SPRY3 and SPRY4) in RAS / MAPK signaling, which leads to an increase in the signaling of this pathway.

On the other hand, Sun et al. (2013) detected a significant overexpression of miR-31 in colorectal cancer. In addition, these authors made an *in silico* identification and an *in vitro* validation of one of the targets of this microRNA, RASA1, which is a regulatory molecule of the RAS-RAF-MAPK pathway. Moreover, Sun et al. (2013) demonstrated that miR-31 can activate the RAS signaling pathway by inhibiting the RASA1 molecule, which would lead to increase cell growth and would facilitate tumorogenesis.

In view of these results, we believe that miR-31 could be linked with the tumoral growth of the ameloblastoma and with the regulation of osteogenesis.

Different studies [36,37] linked the alterations of miR-592 to neoplastic development in humans. Furthermore, its overexpression in colorectal cancer, in which it would promote tumor progression and metastasis, through the FoxO3A target, a transcription factor regulating apoptosis [38]. Liu et al. [39] described this overexpression of miR-592 in colorectal cancer associating it to the size of the tumor, the distance of the metastases and the patient survival. With these data, we consider that alterations in miR-592 expression in ameloblastomas would be mainly linked with tumor growth and progression.

MicroRNA-944 has been associated with the P63 gene, which participates in the process of proliferation and differentiation of keratinocytes [40]. It has been related to the induction of keratins 1 and 10 by inhibiting the expression of ERK signaling and the upregulation of p53 expression [40]. Overexpression of miR-944 is also described in uterine cervix cancer [41], in which it would promote proliferation, migration and cell invasion [41]. Recently, He et al. [42] described its overexpression in breast cancer, suggesting a link with cell proliferation and metastasis. Based on these studies, we believe that the overexpression of miR-944 in ameloblastoma may be related to the processes of epithelial tumor differentiation and neoplastic proliferation.

In our study, we were able to recognize significant differences in miR-489 expression between SA and UA. Although the information on the functions of miR-489 so far is scarce, Schoolmeesters et al. [43] pointed out that this microRNA could regulate the early osteogenic differentiation in human mesenchymal stem cells, and play a critical role in the osteogenic process. In addition, this microRNA has also been identified as a tumor suppressor in hypopharyngeal squamous cell carcinoma [44], lung cancer (NSCLC) [45], and breast cancer [46, 47]. In particular, Patel et al. [47] analyzed the expression profile of deregulated microRNAs in HER2-positive breast cancer cells. Her2 is a receptor tyrosine kinase usually overexpressed in 20–30% of breast cancers and associated with poor prognosis and outcome. These authors observed that miR-489 was underexpressed in this type of cancer, especially through the MAPK pathway, In a later *in vitro* experiment in xenograft mice, they determined that overexpression of this microRNA in HER2-positive breast cancer cells significantly inhibited cell growth and decreased tumorigenicity and tumor growth. In summary, these authors suggest that these results define a double-negative feedback loop involving miR-489 and the HER2-SHP2-MAPK signaling axis that can regulate breast cancer cell proliferation and tumor progression and might have therapeutic relevance for HER2-positive breast cancer [47].

Based on these facts, it could be suggested that the differences found in miR-489 expression between the two types of ameloblastoma could play an important role to explain the different aggressiveness observed in these entities.

The differences in miR-489 expression could be an important element in the differential diagnosis between the two types of ameloblastomas, and therefore, it is essential to confirm these results in a larger set of samples. Although a robust statistical approach was used, there are also some limitations, such as the small number of UA included in the validation phase, or the fact that total RNA was obtained from FFPE samples.

In conclusion, our study identified an apparently specific profile of aberrant expression of microRNAs for the main types of ameloblastoma. These results were validated in an independent set of samples. MicroRNAs differentially expressed in ameloblastomas are related to neoplastic development, osteogenic process and neoplastic differentiation. In addition, we identified a microRNA (miR-489) suggestive of differentiating between solid and unicystic ameloblastomas. Our findings may be useful to open new avenues of study that will allow us to better understand the etiopathogenesis of these neoplasms, as well as to improve their diagnosis and to define more effective treatments.

## Supporting information

S1 Table Study data. (XLSX)
